# Metabolome and transcriptome signatures shed light on the anti-obesity effect of *Polygonatum sibiricum*

**DOI:** 10.3389/fpls.2023.1181861

**Published:** 2023-04-18

**Authors:** Xiaobin Ou, Xiao Wang, Bing Zhao, Yi Zhao, Haiqing Liu, Yuankai Chang, Zhiwei Wang, Wenqi Yang, Xuebin Zhang, Ke Yu

**Affiliations:** ^1^ Gansu Key Laboratory of Protection and Utilization for Biological Resources and Ecological Restoration, College of Life Sciences and Technology, Longdong University, Qingyang, Gansu, China; ^2^ State Key Laboratory of Crop Stress Adaptation and Improvement, Henan Joint International Laboratory for Crop Multi-Omics Research, School of Life Sciences, Henan University, Kaifeng, China; ^3^ School of Life Sciences, Henan University, Kaifeng, China

**Keywords:** multi-omics, metabolome, transcriptome, *Polygonatum sibiricum*, anti-obesity

## Abstract

Obesity has become one of the major threats to human health across the globe. The rhizomes of *Polygonatum sibiricum* have shown promising anti-obesity effect. However, the metabolic and genetic basis mediating this beneficial effect are not fully resolved. It is well known that older rhizomes of *P. sibiricum* exert stronger pharmacological effects. Here, we performed high-resolution metabolome profiling of *P. sibiricum* rhizomes at different growth stages, and identified that three candidate anti-obesity metabolites, namely phloretin, linoleic acid and α-linolenic acid, accumulated more in adult rhizomes. To elucidate the genetic basis controlling the accumulation of these metabolites, we performed transcriptome profiling of rhizomes from juvenile and adult *P. sibiricum*. Through third-generation long-read sequencing, we built a high-quality transcript pool of *P. sibiricum*, and resolved the genetic pathways involved in the biosynthesis and metabolism of phloretin, linoleic acid and α-linolenic acid. Comparative transcriptome analysis revealed altered expression of the genetic pathways in adult rhizomes, which likely lead to higher accumulation of these candidate metabolites. Overall, we identified several metabolic and genetic signatures related to the anti-obesity effect of *P. sibiricum*. The metabolic and transcriptional datasets generated in this work could also facilitate future research on other beneficial effects of this medicinal plant.

## Introduction

*Polygonatum sibiricum* is a monocotyledonous flowering plant in the family Asparagaceae, which is mostly distributed in the Northern Hemisphere, especially in Asia. As a well-known traditional Chinese herb, *P. sibiricum* has been used as medicine or food for over 1500 years (Chen et al., 2015). In the past decades, the medicinal and nutritional values of *P. sibiricum* were investigated and well-documented. Previous studies have reported diverse pharmacological effects of *P. sibiricum*, such as anti-aging, anti-diabetic, anti- inflammatory, and anti-tumor effects (Zhao et al., 2018). These beneficial effects could be attributed to the diverse biologically-active compounds accumulated in the rhizome, such as polysaccharides, alkaloids, saponins, and phenolics (Luo et al., 2022). For example, polysaccharides derived from *P. sibiricum* were shown to significantly alleviate several memory and cognitive impairments as well as aging characteristics in rats fed with the aging reagent D-galactose (Zheng, 2020). Saponins derived from *P. sibiricum* were shown to induce hypoglycemic effects through regulation of gut microbiota in diabetic mice (Luo et al., 2020). However, most research on the pharmacological effects of *P. sibiricum* focused on specialized categories of bioactive compounds, and the genetic factors controlling the biosynthesis and accumulation of these compounds remain largely unexplored.

Obesity significantly contributes to diseases such as type 2 diabetes, stroke and cancer, and has become a major health threat on a global scale, causing severe declines not only in quality of life but also in socio-economic productivity (Bluher, 2019). Thus, prevention of obesity is in urgent need to reduce the adverse impact on both public health and economy. Pharmacological treatments for obesity have been adopted, and several anti-obesity medications have been approved for long-term weight management or under clinical investigations (Heymsfield and Wadden, 2017; Pilitsi et al., 2019). However, due to consideration of various side effects, the willingness for long-term administration of these medications is relatively low. For this reason, researchers kept exploring alternative sources for developing effective and safe anti-obesity agents that could be supplemented *via* daily diet. Previously, it was reported that extracts of *P. sibiricum* rhizomes could significantly ameliorate the obesity induced by high-fat diet in mice (Ko et al., 2015). This anti-obesity effect can be attributed to polysaccharides accumulated in *P. sibiricum*, which could regulate several signaling pathways involved in lipid metabolism, such as adenosine monophosphate-activated protein kinase pathway, to reduce the adverse effects caused by lipid accumulation (Liu et al., 2021; Zeng et al., 2022). Plants produce a wide variety of metabolites and are considered as the most important reservoirs of metabolites on Earth (Fang et al., 2019). Likewise, thousands of bioactive metabolites are simultaneously present in rhizomes of *P. sibiricum*. However, whether rhizomes of *P. sibiricum* contain more metabolites with anti-obesity effects other than polysaccharides remains unknown.

Rhizomes from older *P. sibiricum* often exhibit stronger pharmacological effects than those from younger plants, likely due to the accumulation of bioactive metabolites in rhizomes. Here, we performed high-resolution metabolome analysis and transcriptome profiling in rhizomes harvested at different growth stages in their life cycle, to dissect the metabolic and genetic basis mediating the anti-obesity effect. We found that the rhizomes from adult plants accumulated significantly higher amounts of phloretin, linoleic acid and α-linolenic acid, all of which could potentially exert anti-obesity effect. Moreover, by combining next-generation short-read sequencing and third-generation long-read sequencing of the transcriptome of *P. sibiricum* rhizomes, we identified the differentially expressed genetic pathways responsible for the biosynthesis and accumulation of these metabolites. Our work provides a valuable resource for future studies on the beneficial effects of *P. sibiricum*.

## Results

### High-resolution metabolome profiling of *P. sibiricum* rhizomes

**Figure 1:**
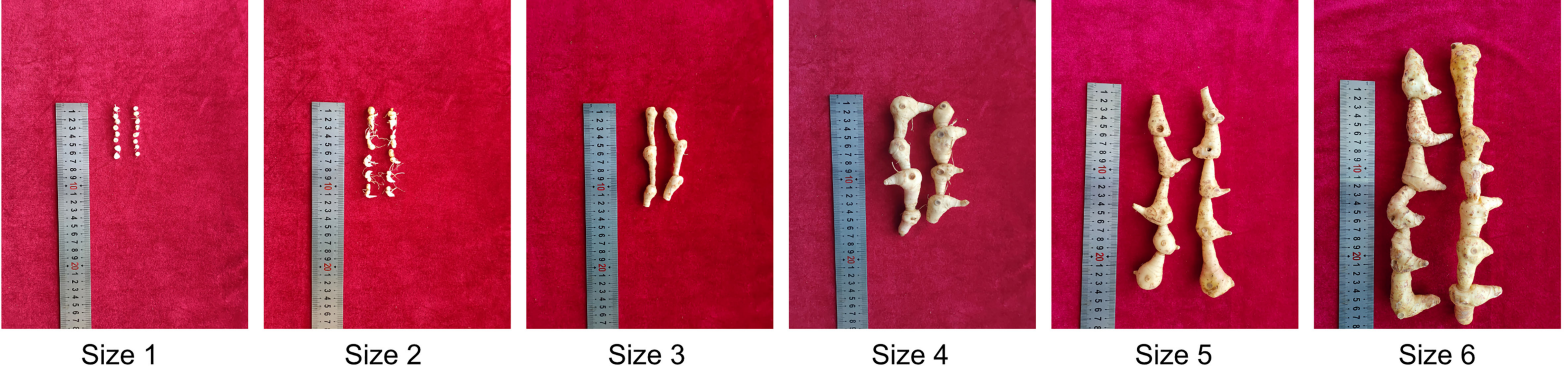
The morphology of rhizomes of *P. sibiricum* at different growth stages. The rhizomes were grouped according to the age.

Six groups of rhizomes representing *P. sibiricum* plants from juvenile to adult growth stages were obtained (Size 1 to 6, **Figure 1**). We extracted the total metabolites from all samples and performed untargeted metabolome analysis by using ultra-high performance liquid chromatography coupled to high-resolution tandem mass spectrometry (UHPLC-MS^2^). An equal volume of each extract was mixed together to make a quality control (QC) sample, which was used to correct the systemic error generated during sample loading as well as batch effect. In total, we identified 29,125 metabolite features from the negative (12,316) and positive (16,809) ion mode for detection. We performed stringent data cleaning in order to get putative metabolites with high-confidence annotations (see methods section). After removal of background noise, 6,618 metabolite features (2,156 from negative mode and 3,962 from positive mode) retained. The peak areas of these metabolite features were normalized by using QC samples. As a result, all QC samples were grouped together as indicated by the principal component analysis (PCA, **Figure S1**), suggesting that the systemic error was largely corrected. Moreover, batch effect was also erased (**Figure S2**). Over 95% putative metabolites have a relative standard deviation (RSD) value that is smaller than 0.3 (**Figure S3**), suggesting consistency and reliability of the metabolome data. We then annotated the 6,618 metabolites features and finally obtained 776 putative metabolites with high-confidence annotations (**Table S1**). Through this high-resolution metabolome profiling strategy, we built a metabolite library of rhizomes from *P. sibiricum* that could be utilized for the following comparative metabolome analysis.

### Comparative metabolome analysis revealed higher accumulation of anti-obesity metabolites in rhizomes of adult *P. sibiricum*

**Figure 2:**
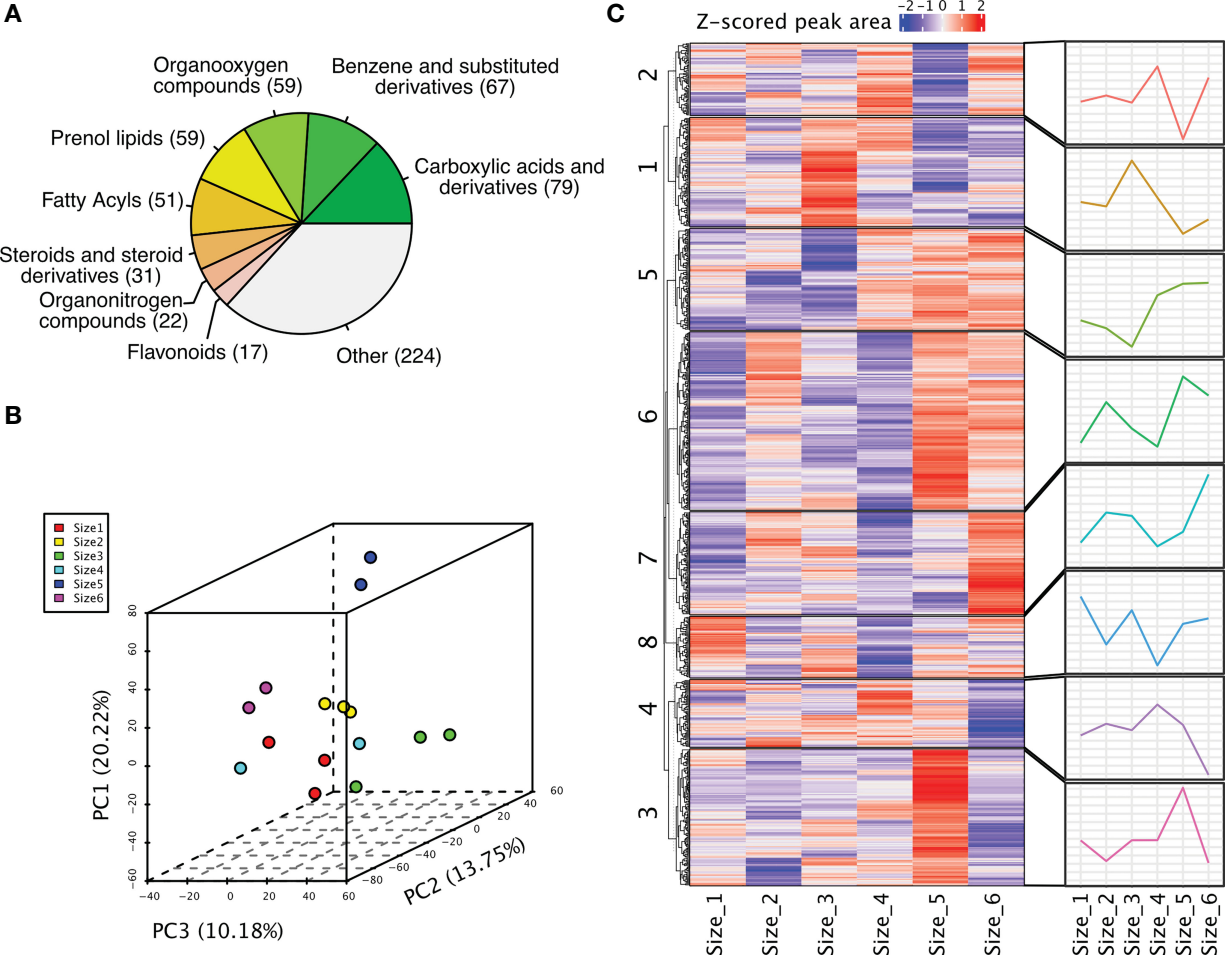
Metabolome profiling of rhizomes from *P. sibiricum* at different growth stages. **(A)** The major classes of metabolites detected in *P. sibiricum* rhizomes. **(B)** Principal component analysis (PCA) of peak areas of metabolites detected in rhizomes from different growth stages. **(C)** K-means clustering of all detected metabolites in rhizomes from different growth stages. The peak areas were used for clustering. The heatmap depicts the relative abundance of each metabolite in different samples, and the line charts depict the accumulation pattern of metabolites in each cluster.

To get a glimpse of the metabolites presented in the rhizomes of *P. sibiricum*, we categorized these metabolites based on their annotations. Major classes of metabolites include carboxylic acids and derivatives (79), benzene and substituted derivatives (67), organooxygen compounds (59), prenol lipids (59), fatty acyls (51), steroids and steroid derivatives (31), organonitrogen compounds (22), and flavonoids (17) (**Figure 2A**, **Table S2**). To investigate whether a clear difference could be detected among rhizomes from different growth stages, we performed PCA of the peak areas of these metabolites. The first three PCs could explain 20.22%, 13.75% and 10.18% of the variation (**Figure 2B**). It appeared that samples of rhizomes from younger *P. sibiricum* (Size 1, 2, 3 and 4) grouped together, while older rhizomes (Size 5 and 6) are more distant from the others in PCA (**Figure 2B**), suggesting that the metabolome profiles of rhizomes from younger *P. sibiricum* are more alike and are different from those from older plants. The metabolome profiles of Size 5 and Size 6 also differed obviously as indicated by the distance of the samples in PCA (**Figure 2B**), suggesting relatively large variations in metabolome profiles at the later growth stages of *P. sibiricum*. We performed K-means clustering to examine the accumulation patterns of these metabolites in different growth stages (**Table S3**). The 776 metabolites could be clustered into 8 clusters, in which clusters 5 and 6 are more abundant in older rhizomes (**Figure 2C**). Moreover, metabolites in clusters 3 and 7 are more abundant in Size 5 or Size 6, respectively (**Figure 2C**). These results suggest a clear metabolic shift between rhizomes from younger and older *P. sibiricum*.

**Figure 3:**
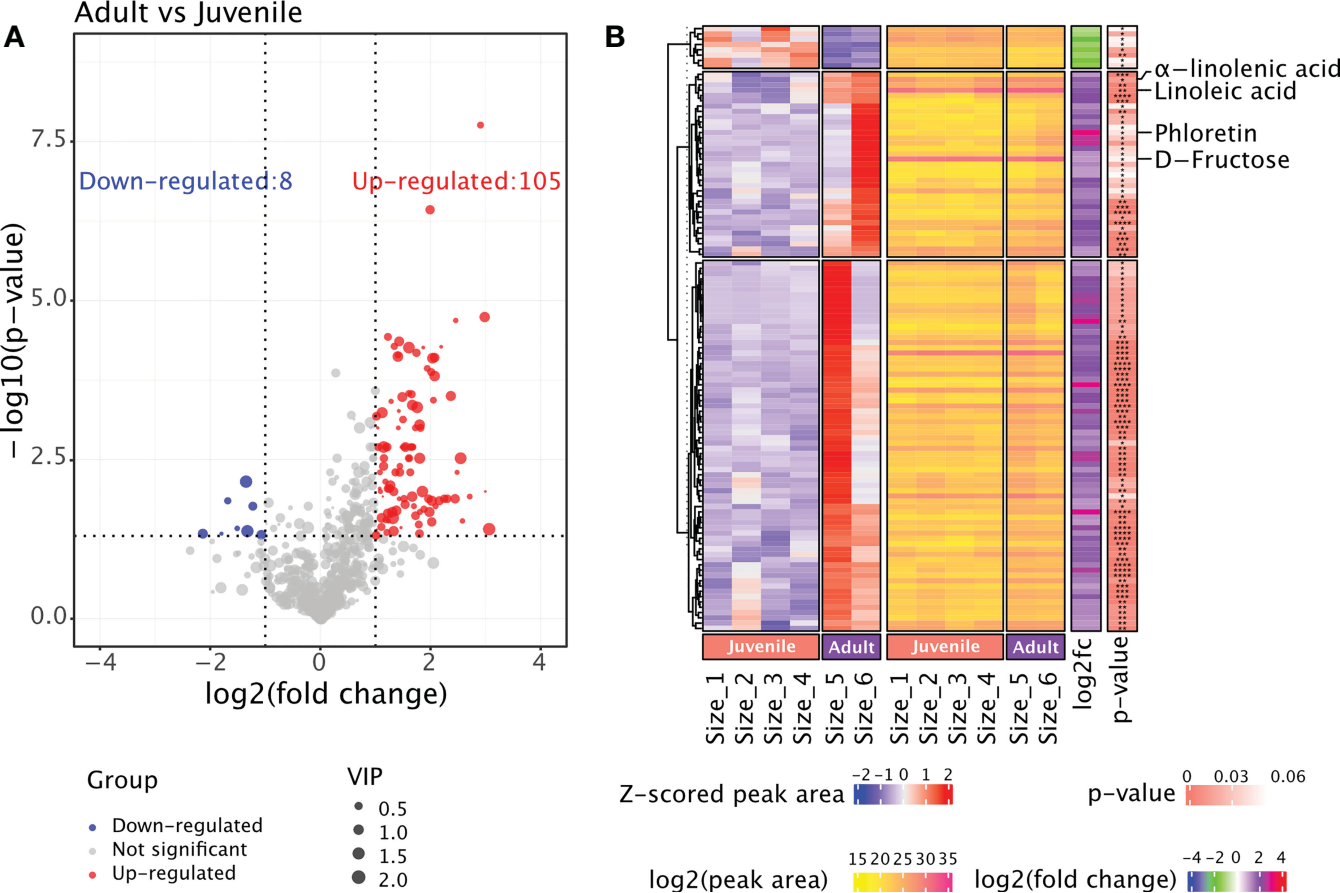
Comparative metabolome analysis of metabolites in rhizomes from *P. sibiricum* at different growth stages. **(A)** Vocano plot of differentially accumulated metabolites (DAMs) in rhizomes from juvenile and adult *P. sibiricum*. Dots in blue represent down-regulated metabolites in adult rhizomes. Dots in red represent up-regulated metabolites in adult rhizomes. Dots in grey represent metabolites with similar abundance in juvenile and adult rhizomes. The size of each dot is determined by variable influence on projection (VIP) score of OPLS-DA analysis. **(B)** Complex heatmaps of DAMs in rhizomes from juvenile and adult *P. sibiricum*. The first heatmap depicts the Z-scored peak areas of DAMs, and the second heatmap depicts the log2 (peak area) values of DAMs. ****, P ≤ 0.0001; ***, 0.0001 < P ≤ 0.001; **, 0.001 < P ≤ 0.01; *, 0.01 < P < 0.05.

The rhizome samples from six groups were regrouped into two: juvenile (Size 1, 2, 3 and 4) and adult (Size 5 and 6). We performed differential analysis to detect metabolites that accumulated differently in juvenile and adult rhizomes (**Table S4**). The results revealed 105 metabolites that are significantly more abundant in adult rhizomes, while the number of metabolites that are less abundant is only eight (**Figure 3A**). We speculated that the bioactive metabolites conferring the anti-obesity effect of *P. sibiricum* are within the 105 metabolites. Therefore, we examined the accumulation patterns of those metabolites and focused on those that accumulated more in both Size 5 and Size 6 (**Figure 3B**). Phloretin, a phenylpropanoid derivative that exhibited the largest difference in abundances between juvenile and adult rhizomes, caught our attention. Previous study suggested that phloretin could significantly prevent obesity and metabolic disorder in mice fed with high-fat diet (Alsanea et al., 2017). In adult rhizomes, the average peak area of phloretin is approximately eight times of that in juvenile rhizomes (**Figure 3B**). Linoleic acid and α-linolenic acid (also known as ω-6 and ω-3 fatty acids, respectively) are two polyunsaturated fatty acids (PUFAs) that are essential for maintaining human health, especially for the prevention of cardiovascular disease (Mozaffarian and Wu, 2011; Farvid et al., 2014), but current evidences suggest they might exhibit distinct effects on obesity (Alvheim et al., 2012; O'Reilly et al., 2020). Nonetheless, both linoleic acid and α-linolenic acid accumulated more in adult rhizomes (**Figure 3B**). In addition, we found that fructose, one of the key intermediate monosaccharides involved in the biosynthesis of polysaccharides that could exert anti-obesity effect, accumulated more in adult rhizomes (**Figure 3B**). Collectively, the comparative metabolome analysis of rhizomes from *P. sibiricum* at different growth stages identified potential anti-obesity metabolites.

### High-fidelity transcriptome profiling of *P. sibiricum* rhizomes

**Figure 4:**
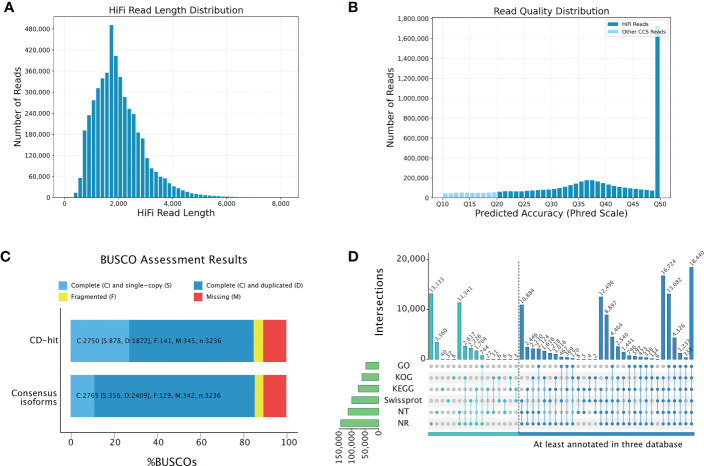
Construction of multi-flux full-length transcriptome of *P. sibiricum*. **(A)** Length distribution of high fidelity (HiFi) reads. **(B)** Quality distribution of HiFi reads. **(C)** BUSCO evaluation of the transcriptome completeness. CD-hit represents non-redundant transcripts. Consensus isoforms represent all detected full-length transcripts. **(D)** Annotation of the full-length nonredundant transcripts according to multiple databases.

Although the potential metabolic basis underlying the anti-obesity effect of *P. sibiricum* was revealed by comparative metabolome analysis, it is not clear that how *P. sibiricum* regulates the biosynthesis and accumulation of these compounds. Therefore, we performed transcriptome analysis to elucidate the genetic basis underlying the anti-obesity effect of *P. sibiricum*. Since the genome of *P. sibiricum* is currently unavailable, we combined long-read third-generation sequencing and short-read next-generation sequencing to examine the transcriptional profile of the rhizomes from juvenile and adult *P. sibiricum*. The ultra-long read length of third-generation sequencing could facilitate the identification of transcripts presented in *P. sibiricum*, which could be utilized as references for mapping of the short-read sequencing data. We obtained 75.66 Gb long-read sequencing data on PacBio Sequel II platform. After removal of adaptors and low-quality reads, 38,696,058 subreads with an N50 of 2,164 bp and an average read length of 1,955 bp retained. After identification of redundant reads and self-correction, we obtained 4,714,912 high-quality reads of insert, which are also referred to as high fidelity (HiFi) reads. These HiFi reads have a read length distribution from 300 bp to 7,000 bp, an average read length of 1,997 bp (**Figure 4A**), and an average Phred scale of Q41 (**Figure 4B**), indicating high data quality and low error rates. Full-length non-chimeric (FLNC) reads accounted for 88.28% of the HiFi reads, with an average read length of 1,947 bp and an N50 of 2,196 bp. After clustering of redundant FLNC reads, polishing for each cluster, and removal of redundant FLNC reads, we obtained 176,643 high-quality non-redundant isoforms. These isoforms constitute a reference transcript pool for mapping of the short-read sequencing data. Finally, we performed BUSCO analysis, and found that 84.95% of the isoforms in this pool were complete (**Figure 4C**, **Tables S5**, **S6**), indicating a fine quality suitable for subsequent functional annotation.

We then annotated the reference transcript pool by using Gene Ontology (GO), Eukaryotic Orthologous Group of proteins (KOG), Kyoto Encyclopedia of Genes and Genomes (KEGG), Swiss-Prot, NCBI nucleotide sequences (Nt) and NCBI non-redundant protein sequences (Nr) databases (**Table S7**). Among 176,643 high-quality non-redundant isoforms, 143,998 isoforms could be annotated by at least one database, while 108,732 isoforms could be annotated by at least three databases (**Figure 4D**). Most isoforms could be matched in Nr database (140,472, 97.56%), of which 87,699 annotated isoforms (60.9%) could match with the annotation of *Asparagus officinalis* (**Table S8**). This result is consistent with a previous report, in which a similar long-read sequencing approach was applied to dissect the transcriptome of *P. sibiricum* (Liao et al., 2021). Meanwhile, 449 isoforms could be matched with the available sequences of 13 known *Polygonatum* species: *Polygonatum verticillatum* (158), *P. sibiricum* (121), *Polygonatum cyrtonema* (53), *Polygonatum multiflorum* (46), *Polygonatum odoratum* (29), *Polygonatum pubescens* (12), *Polygonatum humile* (7), *Polygonatum* sp. JJ-2020 (7), *Polygonatum zanlanscianense* (6), *Polygonatum kingianum* (4) *Polygonatum stenophyllum* (3), *Polygonatum cirrhifolium* (2), and *Polygonatum roseum* (1) (**Table S9**). The sequence similarity ranges from 37.99% to 100%, which is also comparable with previous report (Liao et al., 2021).

We categorized these isoforms based on the annotations by GO, KOG and KEGG databases. By using GO database, 48,043 isoforms were annotated. In Biological Process, most annotated isoforms fall into the following three subcategories: Cellular process, Metabolic process, and Single-organism process. In Molecular Function, most annotated isoforms fall into the following two subcategories: Binding and Catalytic activity (**Figure S4A**). By using KOG database, 62,479 isoforms were annotated into four major categories, which consists of 25 subcategories. In 15,947 isoforms belonging to Cellular Processes and Signaling, most isoforms fall into the following three subcategories: Signal transduction mechanisms, Posttranslational modification, protein turnover, chaperones, and Intracellular trafficking, secretion, and vesicular transport. In 10,589 isoforms belonging to Metabolism, most isoforms fall into the following six subcategories: Carbohydrate transport and metabolism, Energy production and conversion, Lipid transport and metabolism, Amino acid transport and metabolism, Secondary metabolites biosynthesis, transport and catabolism, and Inorganic ion transport and metabolism (**Figure S4B**). By using KEGG database, 74,622 isoforms were annotated into five major categories: Cellular processes, Environmental information Processing, Genetic information processing, Metabolism, and Organismal systems. Among the 19 subcategories, a significant number of isoforms fall into metabolism of primary and secondary metabolites (**Figure S4C**).

Finally, we predicted the coding sequences of the 177,643 isoforms in this reference transcript pool, and found that 128,026 isoforms contain a coding sequence, which mostly ranges from 300 to 4,000 nt (**Figure S5A**). Moreover, we identified 73,175 isoforms matching features of lncRNA (mostly ranges from 300 to 4,000 nt), of which 39,867 isoforms contain a coding sequence (**Figures S5B, C**). Together, these results provide a high-quality transcript pool that could be utilized for the following comparative transcriptome analysis.

### Comparative transcriptome analysis revealed higher expression of biosynthetic pathways related to anti-obesity metabolites in rhizomes of adult *P. sibiricum*

**Figure 5:**
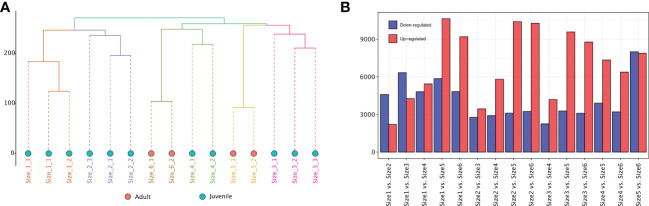
Transcriptome analysis of rhizomes from *P. sibiricum* at different growth stages. **(A)** Hierarchical clustering of different rhizomes using the FPKM values of the transcriptome profiles. The dots in salmon represent adult rhizomes. The dots in turquoise represent juvenile rhizomes. **(B)** The number of up- and down-regulated differentially expressed transcripts (DETs) in the different combinations for comparison.

To identify the biosynthetic pathways related to potential anti-obesity metabolites in rhizomes of adult *P. sibiricum*, we mapped the short-read sequencing data to the reference transcript pool. Mapping resulted in average of 78.25 Mb data for each sample, and the average coverage of the reference was 91.55% (**Table S10**). The rhizomes from juvenile and adult *P. sibiricum* were organized according to hierarchical clustering of the FPKM values, and the biological replicates of each group were clustered together (**Figure 5A**). Correlation analysis suggested that rhizomes from adult *P. sibiricum* (Size 5 and 6) are more distinct from the rest of the samples, while the difference between Size 5 and Size 6 is also relatively large (**Figure S6A**), which is consistent with the metabolome analysis. PCA using FPKM values of the transcriptome profiles revealed similar results (**Figure S6B**). We then performed pair-wise differential analysis to identify the differentially expressed transcripts (DETs) between different groups. In general, adult groups (Size 5 and 6) clearly have more DETs when compared with the juvenile groups (Size 1, 2, 3 and 4, **Figure 5B**). These results suggest a clear difference in the transcriptomes of rhizomes from juvenile and adult *P. sibiricum*.

**Figure 6:**
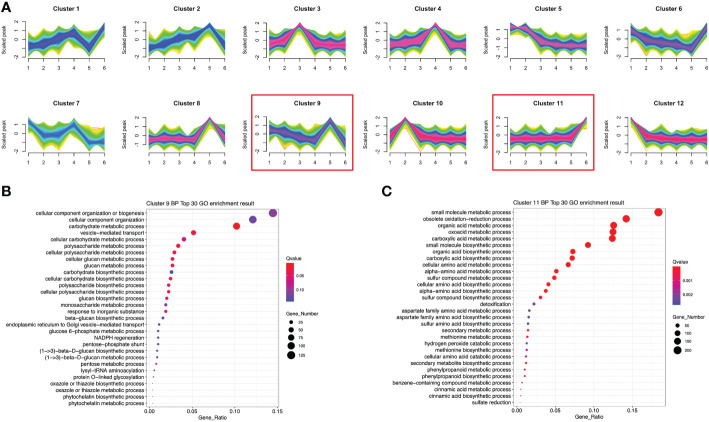
Temporal and functional analysis of differentially expressed transcripts (DETs) among rhizomes from *P. sibiricum* at different growth stages. **(A)** The 12 clusters of DETs showing differential expression patterns in different groups of rhizomes. **(B)** GO enrichment analysis of DETs in cluster 9. **(C)** GO enrichment analysis of DETs in cluster 11. In **(B)** and **(C)**, the color of the dot is determined by the q-value of enrichment analysis, and the size of dot if determined by the number of DETs in each GO term.

To investigate the expression patterns of these DETs in *P. sibiricum* at different growth stages, we performed temporal analysis of all the DETs identified in pair-wise differential analysis. These DETs were clustered into 12 clusters with variable expression patterns (**Figure 6A**). Since we have identified several potential anti-obesity metabolites with higher accumulation in rhizomes from adult *P. sibiricum*, we focused on the DETs that showed higher expression in adult groups (Size 5 and 6). Among 12 clusters, transcripts in clusters 2, 8 and 9 showed highest expression in Size 5, while transcripts in clusters 1, 6 and 11 showed highest expression in Size 6 (**Figure 6A**). We performed GO enrichment analysis of the transcripts in those clusters. As a result, the pathways related to biosynthesis and metabolism of carbohydrates and polysaccharides were enriched in cluster 9 (**Figure 6B**), while pathways related to biosynthesis of primary and secondary metabolites, such as phenylpropanoids, were enriched in cluster 11 (**Figure 6C**). Moreover, KEGG analysis revealed that α-linolenic acid metabolism is significantly enriched in cluster 11 (**Figure S7**). These results, in combination with the metabolome analysis, suggested that in adult rhizomes, the biosynthesis and metabolism of phenylpropanoids, unsaturated fatty acids and polysaccharides are more active than juvenile rhizomes.

### Dissection of biosynthetic pathways of anti-obesity metabolites in *P. sibiricum*

**Figure 7:**
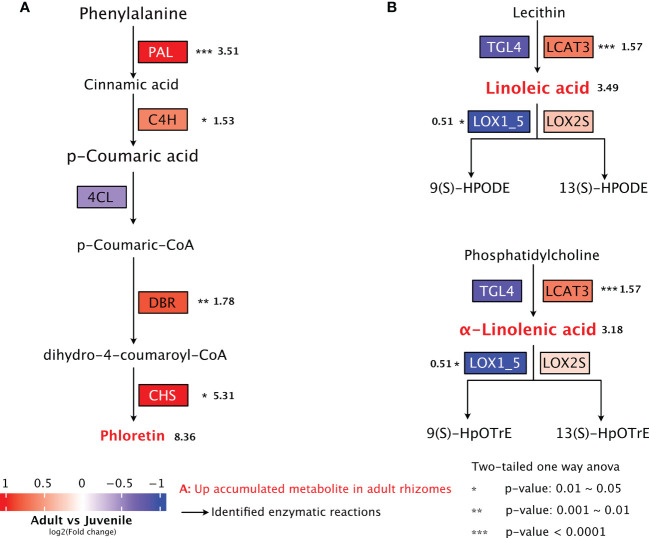
Biosynthetic pathways of potential anti-obesity metabolites in rhizomes of *P. sibiricum*. **(A)** Biosynthesis of phloretin. **(B)** Biosynthesis and metabolism of linoleic acid and α-linolenic acid. The metabolites with higher accumulation in adult rhizomes are colored in red. Asterisks indicate significant difference in the gene expression between juvenile and adult rhizomes. Numbers (in bold) next to asterisks or metabolites are fold changes of the expression levels of indicated genes (adult/juvenile) or fold changes of peak areas of metabolites colored in red (adult/juvenile). The arrows represent the enzymatic reactions.

By combining the metabolome and transcriptome analysis, we investigated the expression of key genes involved in the biosynthetic pathways of metabolites with potential anti-obesity effect in *P. sibiricum*. The biosynthesis of phloretin, a dihydrochalcone, was mostly studied in apple, which involves phenylpropanoid pathway and flavonoid pathway (Dare et al., 2013; Yahyaa et al., 2017). In phenylpropanoid pathway, phenylalanine is deaminated into cinnamic acid by phenylalanine ammonia-lyase (PAL), then hydroxylated into *p*-coumaric acid by cinnamate 4-hydroxylase (C4H), and finally converted into *p*-coumaroyl CoA by 4-coumarate:CoA ligase (4CL) (Fraser and Chapple, 2011). As one of the most important phenylpropanoid intermediate in plants, *p*-coumaroyl CoA enters the flavonoid pathway through the action of double bond reductase (DBR), which catalyzes the conversion of *p*-coumaryl-CoA to *p*-dihydrocoumaryl-CoA (Dare et al., 2013; Yahyaa et al., 2017). Subsequently, *p*-dihydrocoumaryl-CoA is converted into phloretin by chalcone synthase (CHS), the rate limiting enzyme of flavonoid biosynthesis (Dare et al., 2013; Yahyaa et al., 2017; Zhang et al., 2017). We found that in rhizomes from adult *P. sibiricum*, the expressions of *PAL*, *C4H*, *DBR* and *CHS* are all significantly higher than those in juvenile rhizomes (**Figure 7A**, **Table S11**), which likely leads to the higher accumulation of phloretin in adult rhizomes.

According to KEGG database (map00591, Linoleic acid metabolism), linoleic acid could be produced from lecithin by the action of phospholipase (enzyme 3.1.1.4 or 3.1.1.32). We identified two phospholipases in *P. sibiricum:* triacylglycerol lipase 4 (TGL4, enzyme 3.1.1.4) and phospholipase A1 (LCAT3, enzyme 3.1.1.32) (**Table S11**). Linoleic acid could be dioxygenated into 9(S)-HPODE or 13(S)-HPODE by lipoxygenases (LOX), which refer to LOX1_5 and LOX2S in *P. sibiricum* (**Table S11**). Similarly, TGL4 and LCAT3 could catalyze the production of α-linolenic acid from phosphatidylcholine (map00592, α-Linolenic acid metabolism), while LOX1_5 and LOX2S could dioxygenate α-linolenic acid into 9(S)-HPODEm or 13(S)-HpOTrE. We found that the expression of *LCAT3* is significantly higher, and the expression of *LOX1_5* is significantly lower in adult rhizomes (**Figure 7B**). Meanwhile, no significant difference was detected in the expression of *TGL4* or *LOX2s* in juvenile and adult rhizomes (**Figure 7B**). These results suggest more active biosynthesis and less active metabolism of linoleic acid and α-linolenic acid in adult rhizomes, which likely lead to the higher accumulation of both PUFAs.

**Figure 8:**
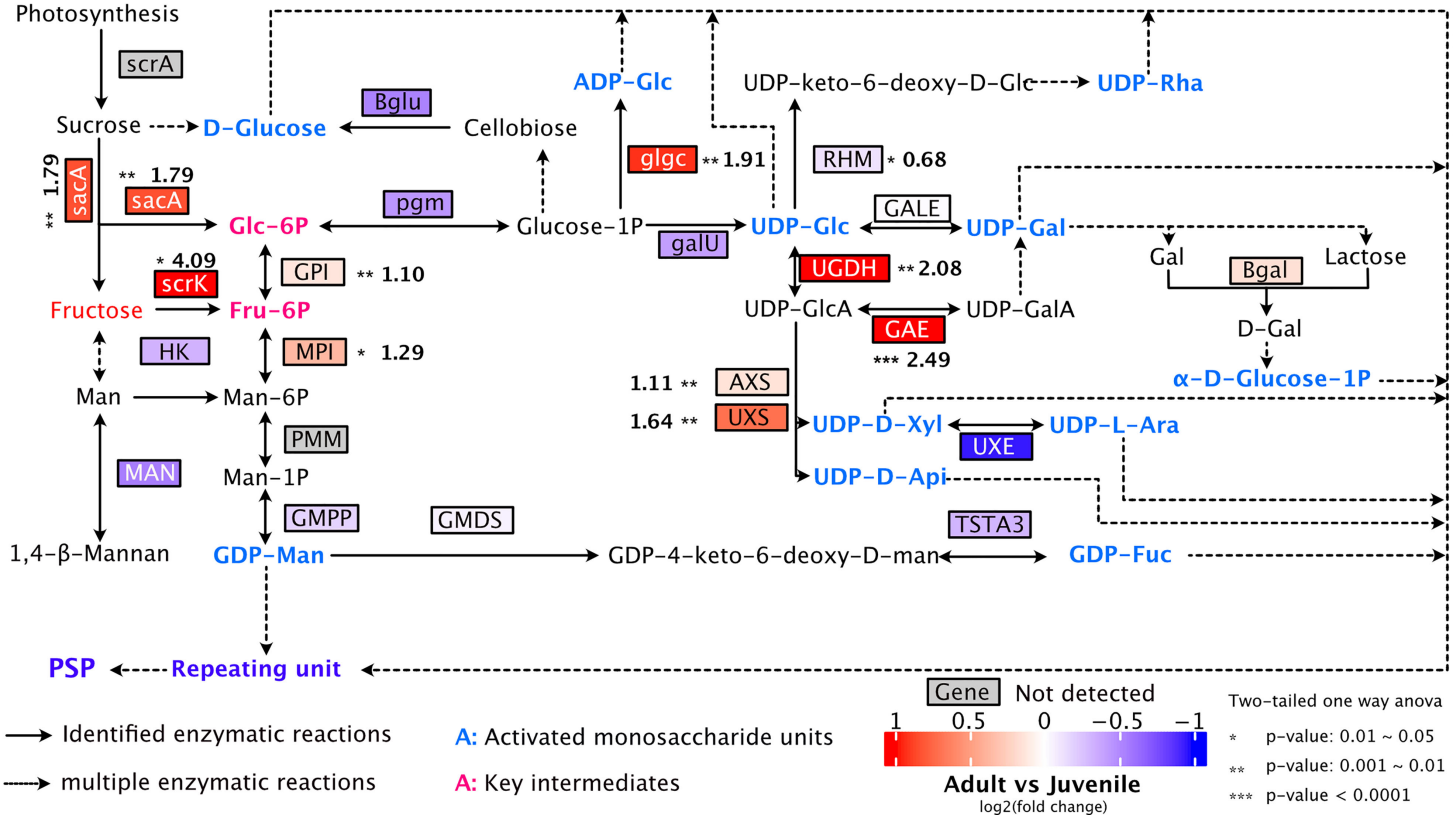
Proposed pathway for polysaccharide biosynthesis in *P. sibiricum*. The solid arrows represent identified enzymatic reactions, and the dashed arrows represent multiple-step enzymatic reactions. Fructose is the metabolite with higher accumulation in adult rhizomes. Key intermediates are colored in rose red and activated monosaccharide units are colored in blue. Asterisks indicate significant difference in the gene expression between juvenile and adult rhizomes. Numbers (in bold) next to asterisks are fold changes of the expression levels of indicated genes (adult/juvenile).

The polysaccharides of *P. sibiricum* have shown promising anti-obesity effect (Ko et al., 2015; Liu et al., 2021; Zeng et al., 2022). Although we could not determine the level of polysaccharides due to limitation of analytical methods adopted here, we constituted the biosynthetic pathway of polysaccharides based on the transcriptome data, and examined the expression patterns of key transcripts in both juvenile and adult rhizomes (**Figure 8**). The pathway depicted here is comparable with proposed pathways of polysaccharides in *P. sibiricum* by two previous reports (Wang et al., 2017; Feng et al., 2022). We found that the expressions of transcripts encoding the following enzymes were significantly higher in rhizomes from adult rhizomes: sacA (β-fructofuranosidase), scrK (fructokinase), GPI (glucose-6-phosphate isomerase), MPI (mannose-6-phosphate isomerase), glgC (Glucose-1-phosphate adenylyltransferase), UGDH (UDP-glucose dehydrogenase), GAE (UDP-glucuronate 4-epimerase), AXS (UDP-apiose/xylose synthase), UXS (UDP-glucuronate decarboxylase) (**Figure 8**, **Table S11**). As a result of the elevated expressions of those enzymes, various monosaccharide units that serve as the building blocks of polysaccharides are activated in adult rhizomes (**Figure 8**), which likely lead to higher accumulation of polysaccharides.

## Discussion

Obesity has become one of the major causes of shortened life expectancy (Fontaine et al., 2003). The development of obesity is complicated. Unhealthy expansion of visceral adipose tissue is the key to the progression of obesity, which is accompanied by increased generation of reactive oxygen species (ROS) and release of pro-inflammatory factors such as adipokines (Wisse, 2004; McMurray et al., 2016). These two factors also promote the progression of obesity (Furukawa et al., 2004). Moreover, oxidative stress and inflammation significantly contribute to metabolic disorders, increasing the risk of other obesity-related diseases, such as diabetes, hypertension, etc (Wisse, 2004). The increasing prevalence of obesity and growing costs for the treatment urge for safe and effective anti-obesity measures. Previous reports indicate promising effects of *P. sibiricum* extracts in the prevention of obesity in mice fed with high-fat diet (Ko et al., 2015; Liu et al., 2021; Zeng et al., 2022). Apart from previously identified polysaccharides in *P. sibiricum* rhizomes, here we identified several candidate anti-obesity metabolites, including a phenylpropanoid derivative (phloretin) and two PUFAs (linoleic acid and α-linolenic acid). These metabolites were found accumulated more in adult rhizomes. Since *P. sibiricum* is commonly consumed as medicine or food, it would serve as an important daily diet with significant potential in preventing weight gain.

Phenylpropanoids represent an array of metabolites synthesized through phenylalanine, and are involved in many aspects of plant development and plant defense (Fraser and Chapple, 2011). A for human health, the medicinal benefits of phenylpropanoids have been gradually recognized (Ververidis et al., 2007). Phloretin is a natural dihydroxychalcone flavonoid that is mostly found in fruits such as apple and strawberry (Hilt et al., 2003; Tsao et al., 2003). It was shown that phloretin could prevent weight gain of obese mice fed with high-fat diet (Alsanea et al., 2017). It significantly blocked several pathological events that are associated with diet-induced obesity in mice, such as lipid accumulation, development of fatty liver, and decreased insulin sensitivity (Alsanea et al., 2017). Moreover, supplement of phloretin suppressed the expression of pro-inflammatory genes (Alsanea et al., 2017). Like other flavonoids, phloretin possesses strong antioxidant and anti-inflammatory activity (Behzad et al., 2017), which likely explains the anti-obesity effect of phloretin.

Linoleic acid and α-linolenic acid are two essential PUFAs that cannot be synthesized in the body (Spector and Kim, 2015). Linoleic acid is abundantly available in vegetable oils, while α-linolenic acid is abundantly available in fish oils, as well as oils derived from the seeds of some plants such as flaxseed and walnuts. Replacing saturated fatty acid with PUFAs in daily diet has been shown to reduce the risks of several diseases such as coronary artery diseases or type 2 diabetes (Mozaffarian et al., 2010; Rajaram, 2014; Wang et al., 2016). The anti-obesity effect of these two PUFAs, however, have been under debate. Both linoleic acid and α-linolenic acid were shown to prevent the development of type 2 diabetes in obese population (Zhuang et al., 2018), and to maintain insulin resistance in obese rats (Matravadia et al., 2014). A number of studies suggested that high intakes of α-linolenic acid could alleviate the metabolic syndrome and reduce weight gain (Murase et al., 2002; Baxheinrich et al., 2012; Egert et al., 2014), but no clear evidence suggested similar effect of linoleic acid. On the other hand, the conjugated form of linoleic acid, which is mostly found in dairy and beef, has shown promising effects in weight control (Kamphuis et al., 2003; Chen et al., 2012). Interestingly, selected members of the microbiota residing in human gut could facilitate the conversion of linoleic acid to conjugated linoleic acid (Devillard et al., 2007; Huyan et al., 2022), suggesting a potential of linoleic acid in the prevention of weight gain.

The complexity of *P. sibiricum* metabolome profile makes it challenging to fully resolve the potential metabolic basis linking the genotypes to the beneficial effects. Untargeted metabolomics aims to identify the vast majority of metabolites in the specimen, and has been widely used in plant metabolite profiling to explore the mechanisms causing particular phenotypes. By using untargeted metabolomics, a recent report identified that a number of differentially accumulated metabolites account for the different beneficial health potentials and flavor of two types of *P. sibiricum* rhizomes (Luo et al., 2022). Besides, combining this approach with high-throughput transcriptome profiling could not only provide detailed description of the gene-to-metabolite network, but also enable the identification of novel genes involved in the regulation of metabolic features. Although the reference genome of *P. sibiricum* is currently unavailable, long-read transcriptome sequencing have been used to build reference transcripts for the identification of genes involved in the release of seed dormancy (Liao et al., 2021). The metabolite library and transcript pool that we built from untargeted high-resolution metabolome analysis and long-read transcriptome sequencing also enabled us to identify the genes/transcripts responsible for the accumulation of potential anti-obesity metabolites in adult rhizomes.

In summary, through a multi-omics approach, we uncovered a number of metabolic and genetic signatures in *P. sibiricum* that are associated with its anti-obesity effect. The multi-omics data provided here could also serve as valuable sources for future studies of other beneficial effects of *P. sibiricum*.

## Materials and methods

### Metabolite extraction

The rhizomes of *P. sibiricum* that were at different growth stages were harvested, flash frozen in liquid nitrogen, and stored under -80°C until further processing. For metabolite extraction, the rhizomes were ground into fine powder, mixed with 80% HPLC grade methanol containing 1 μM chrysin as the internal standard, and incubated overnight. The ratio of the fresh weight to the volume of extraction solution was kept as 0.1 g/ml. After overnight extraction, the mixture was centrifuged at 13000 rpm for 30 min at 4°C, and the supernatants were filtered through a 0.22 μm filter before loaded into injection vials.

### Metabolite detection

Untargeted metabolome analysis using UHPLC-MS^2^ was performed as described previously (Yu et al., 2022).

### Metabolome data analysis

The metabolomics data was processed by using tidyMass (Shen et al., 2022). In detail, the raw data was converted to.mzXML and.mgf format by msConvert (Adusumilli and Mallick, 2017). Features with a retention time between 1 ~ 18 min were kept. TidyMass with default parameters was used to select the peaks. These steps resulted in the original tidyMass mass_dataset object. To obtain the accurate metabolites accumulation matrix, data cleaning was performed. First, the missing values in each sample were detected. Features with a missing value rate over 20% in all QC samples were considered as noise and removed from the mass_dataset. Second, the missing values were imputed by k-nearest neighbors’ algorithm (k-NN). At last, the systemic errors and batch effects were corrected by support vector regression, a QC-based normalization method (Shen et al., 2016). To annotate the detected features, the MS1 or MS2 information were compared with the public databases, including MS2 databse: MassBank, MassBank of North America (MoNA), Human Metabolome Database (HMDB), and Vaniya/Fiehn Natural Products Library, and MS1 database: KNApSAcK, KEGG and PlantCyc. In addition, MetDNA2 was used to annotate unknown metabolites (Zhou et al., 2022). To reduce the redundant annotation information, the following strategies were applied. For features which matched with MS2 database, all adduct model annotations were retained. For the remaining features, adduct model “[M+H]+” in positive model and adduct model “[M-H]-” in negative model were retained. For features that were annotated as multiple compounds, only the compound with the highest total match score was kept. For multiple features annotated to the same compound, only the feature with the highest total match score was kept. Classification of the metabolites was performed using ClassyFire (Djoumbou Feunang et al., 2016).

### Library preparation for transcriptome sequencing

Total RNA was extracted by using RNAprep Pure Plant Kit (Tiangen Biotech). The integrity and concentration of RNA were assessed using Agilent 2100 Bioanalyser (Agilent Technologies) and NanoDrop 2000 Spectrophotometers (Thermo Fisher Scientific), respectively. For long-read third-generation sequencing, a bulked RNA sample was used. Libraries for transcriptome sequencing were constructed by Berry Genomics. PacBio RS II (Pacific Biosciences) and Illumina NovaSeq 6000 (Illumina) platforms were used for long-read third-generation sequencing and short-read next-generation sequencing, respectively.

### Long-read transcriptome sequencing and data analysis

The long-read transcriptome sequencing was performed by Berry Genomics. First, the low-quality reads and adaptor were removed from the raw reads, which yielded the subreads. Reads of insert (ROI) were retrieved from self-correcting subreads according the following criteria: full passes ≥ 1 and sequence accuracy ≥ 0.9. ROI containing both the 5’ and 3’ primer sequences and a ploy (A) tail were considered to be full-length non-chimeric (FLNC) reads. The FLNC reads were clustered using the iterative isoform-clustering algorithm to *de novo* predict high- and low-quality isoforms. Sequence consistency was corrected by an in-house polish program. Finally, non-redundant isoforms were obtained by using CD-hits (Fu et al., 2012), which constitute the transcript pool for mapping of short-read sequencing data. The completeness of full-length transcriptome was evaluated against *Liliopsida* datasets by BUSCO software (version 5.4.3) (Manni et al., 2021). To annotate the high-quality non-redundant isoforms, they were aligned to GO, KOG, KEGG, Swiss-Prot, Nt and Nr databases by using BLAST (version 2.2.26).

### Short-read transcriptome sequencing and data analysis

The short-read transcriptome sequencing was performed by Berry Genomics. The quality control of raw reads were performed by using FastQC (version 0.11.9), and the reads were trimmed by using Trimmomatic (version 0.39) (Bolger et al., 2014). The clean reads were then mapped to the full-length transcript pool generated through long-read transcriptome sequencing by using Bowtie2 (version 2.1.0) (Langmead and Salzberg, 2012). The abundance of transcripts was calculated and normalized to fragments per kilobase of exon model per million mapped fragments (FPKM) by using RESM (version 1.2.15) (Li and Dewey, 2011). Pairwise differential analysis of expressed transcripts was performed by using DESeq2 (version 1.34.0) with the cutoff setting as P-value < 0.05 and absolute log2-transformed (fold-change) > 1 (Love et al., 2014). Time series analysis of differentially expressed transcripts (DETs) was performed by using Mfuzz (version 2.54.0) (Kumar and E.F, 2007). GO and KEGG enrichment analyses were performed by using TBtools (version 1.1043) (Chen et al., 2020). To evaluate the significance of genes involved in biosynthesis and metabolism of phloretin and PUFAs, DETs between juvenile and adult rhizomes that were annotated as the corresponding gene were collected as a gene set (**Table S11**). Here, transcripts with an identified E value < 1E-100 in at least 2 of the Nr, Nt and SwissProt database were kept. Second, the average FPKM of each gene set was marked as the in-silico expression level, and two-tailed one-way ANOVA analysis was performed to examine the difference in expression between juvenile and adult rhizomes.

## Data availability statement

The datasets presented in this study can be found in online repositories. The names of the repository/repositories and accession number(s) can be found below: https://www.ncbi.nlm.nih.gov/, PRJNA941943.

## Author contributions

XO conceived the project. XO and XW performed most of the experiments and analyzed the data. BZ performed metabolome analysis. YZ, HL, ZW, WY and YC assisted in experiments. XO, XZ and KY designed the experiments, analyzed the data and wrote the manuscript. All authors contributed to the article and approved the submitted version.
