# Supplementary figures and images for: Metabolome and transcriptome signatures shed light on the anti-obesity effect of Polygonatum sibiricum
Source: Front Plant Sci. 2023 Apr 18;14:1181861. doi: 10.3389/fpls.2023.1181861 (PMC10151794; doi:10.3389/fpls.2023.1181861)

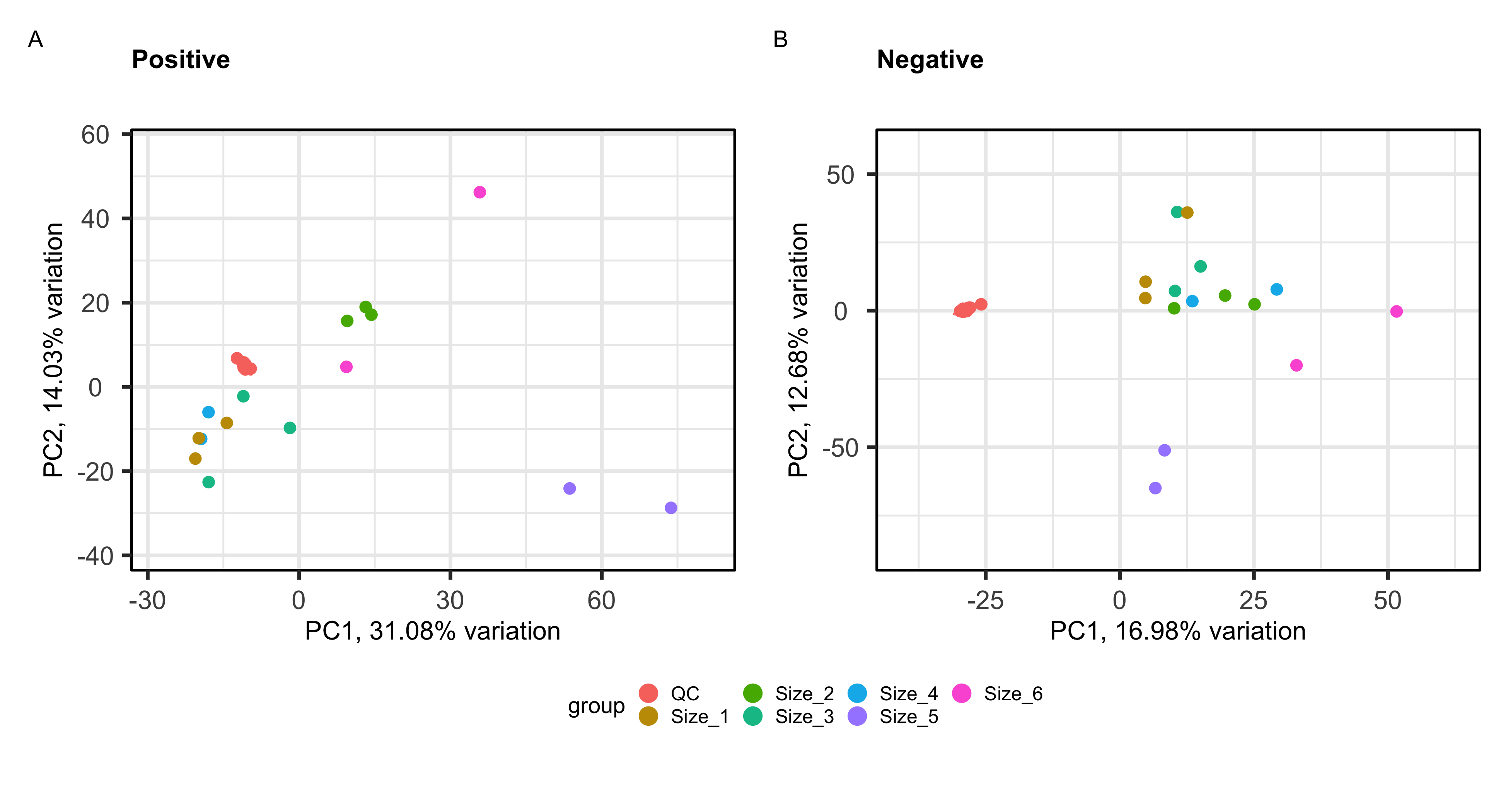

Supplement: Supplementary file 1 [file Image_1.jpg]

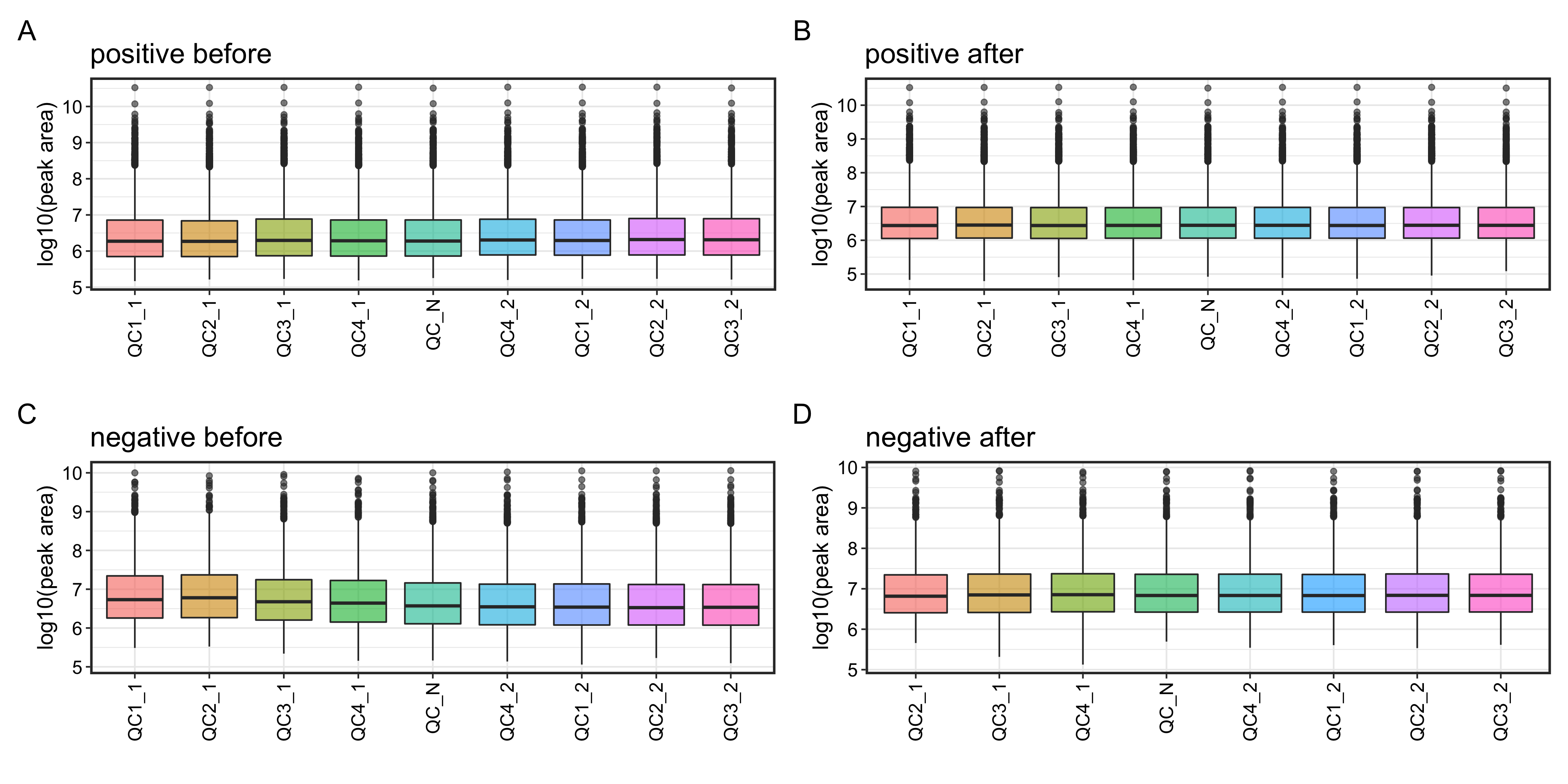

Supplement: Supplementary file 2 [file Image_2.jpg]

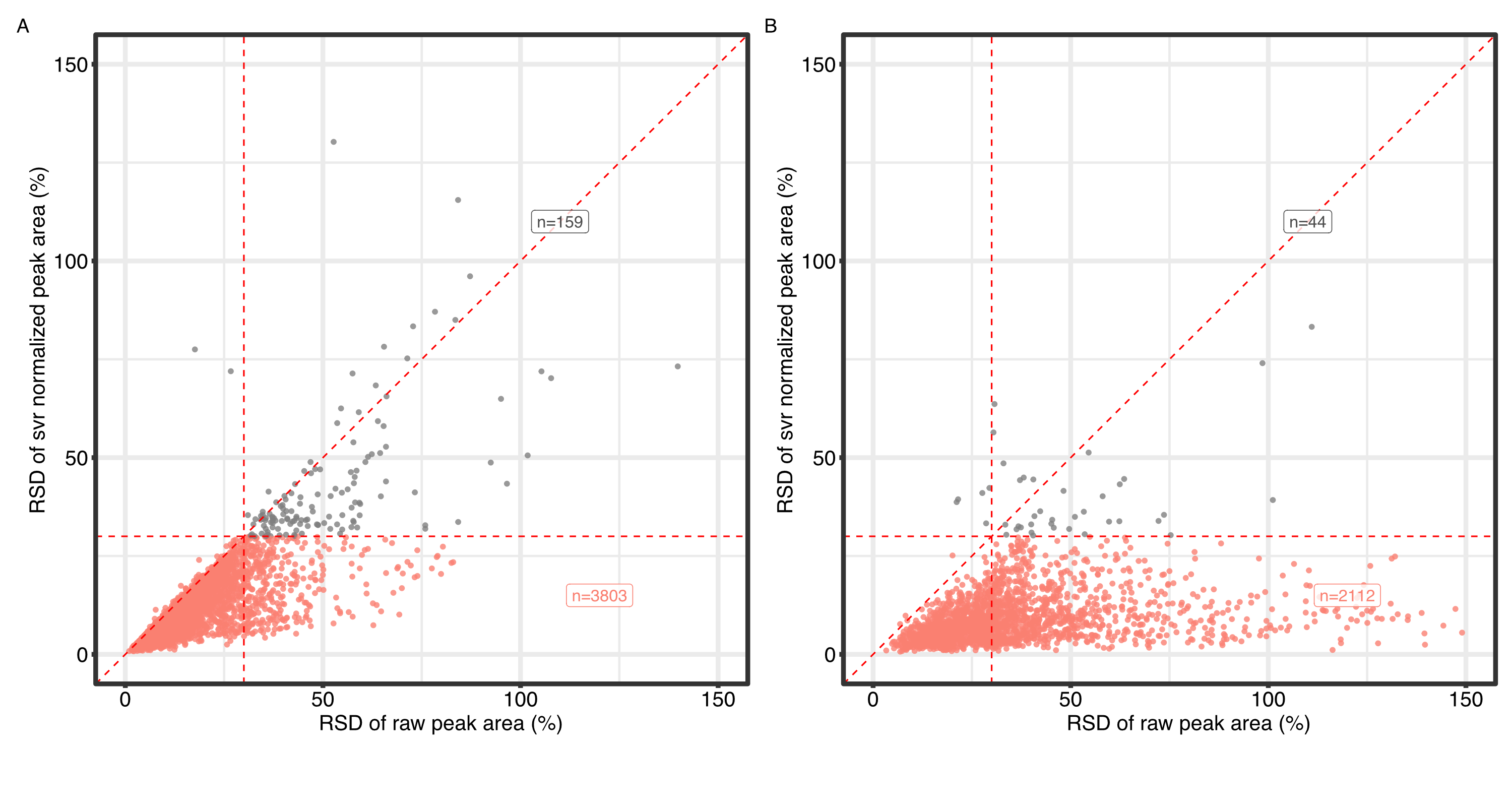

Supplement: Supplementary file 3 [file Image_3.jpg]

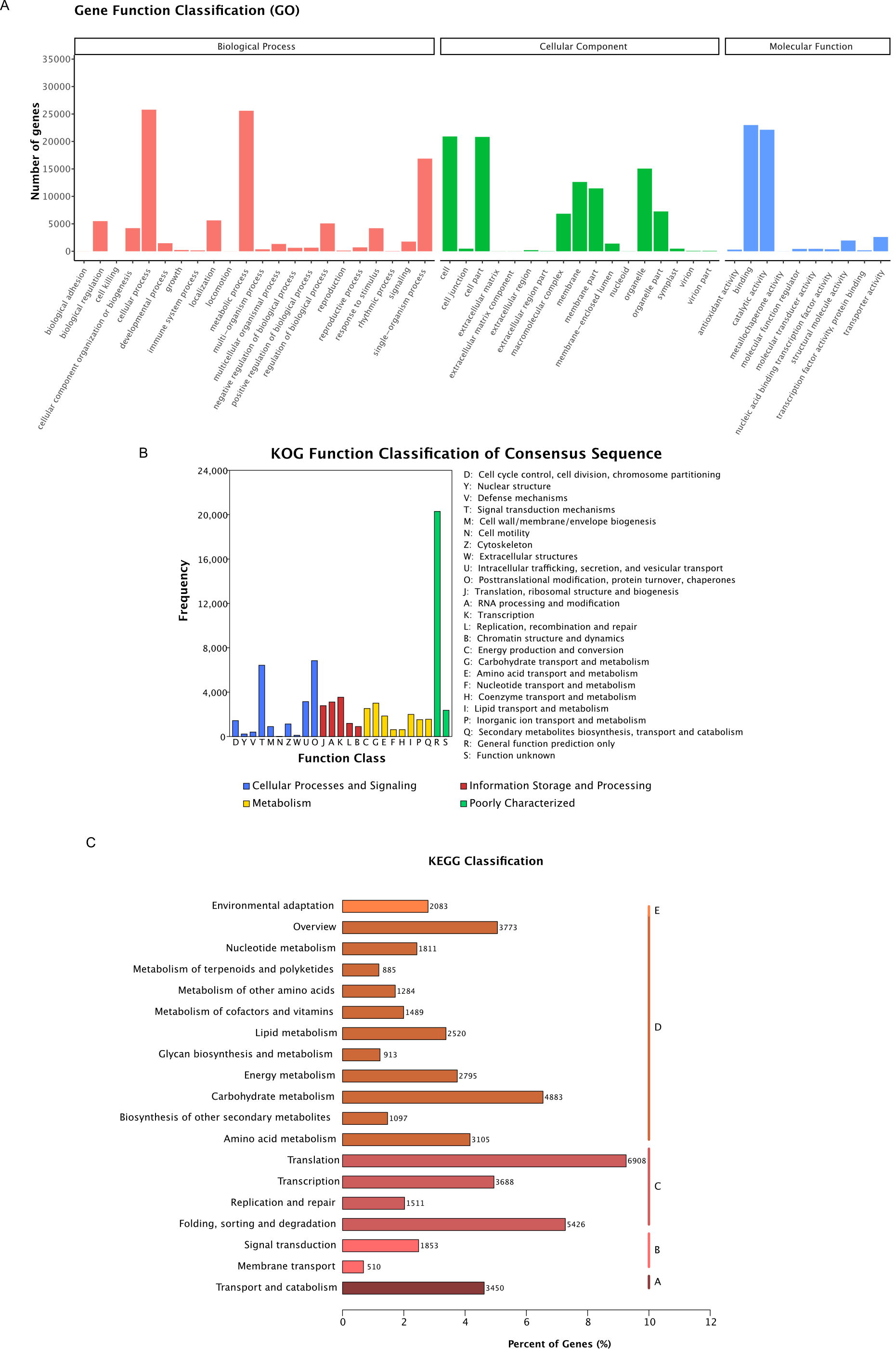

Supplement: Supplementary file 4 [file Image_4.jpg]

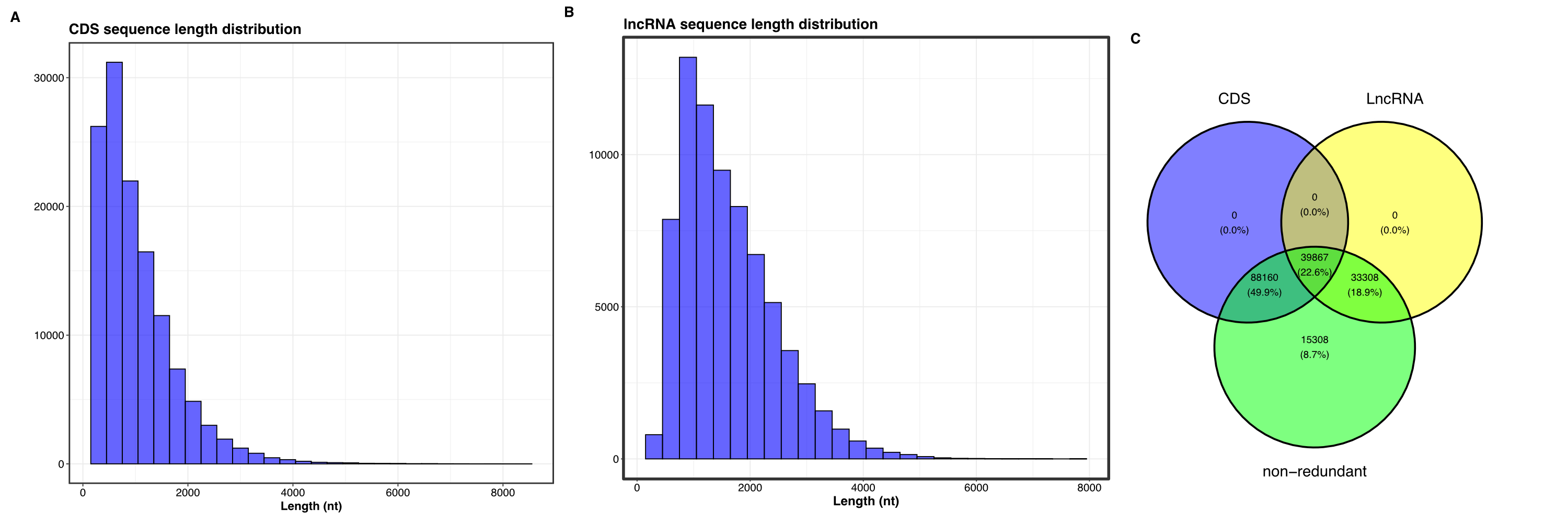

Supplement: Supplementary file 5 [file Image_5.jpg]

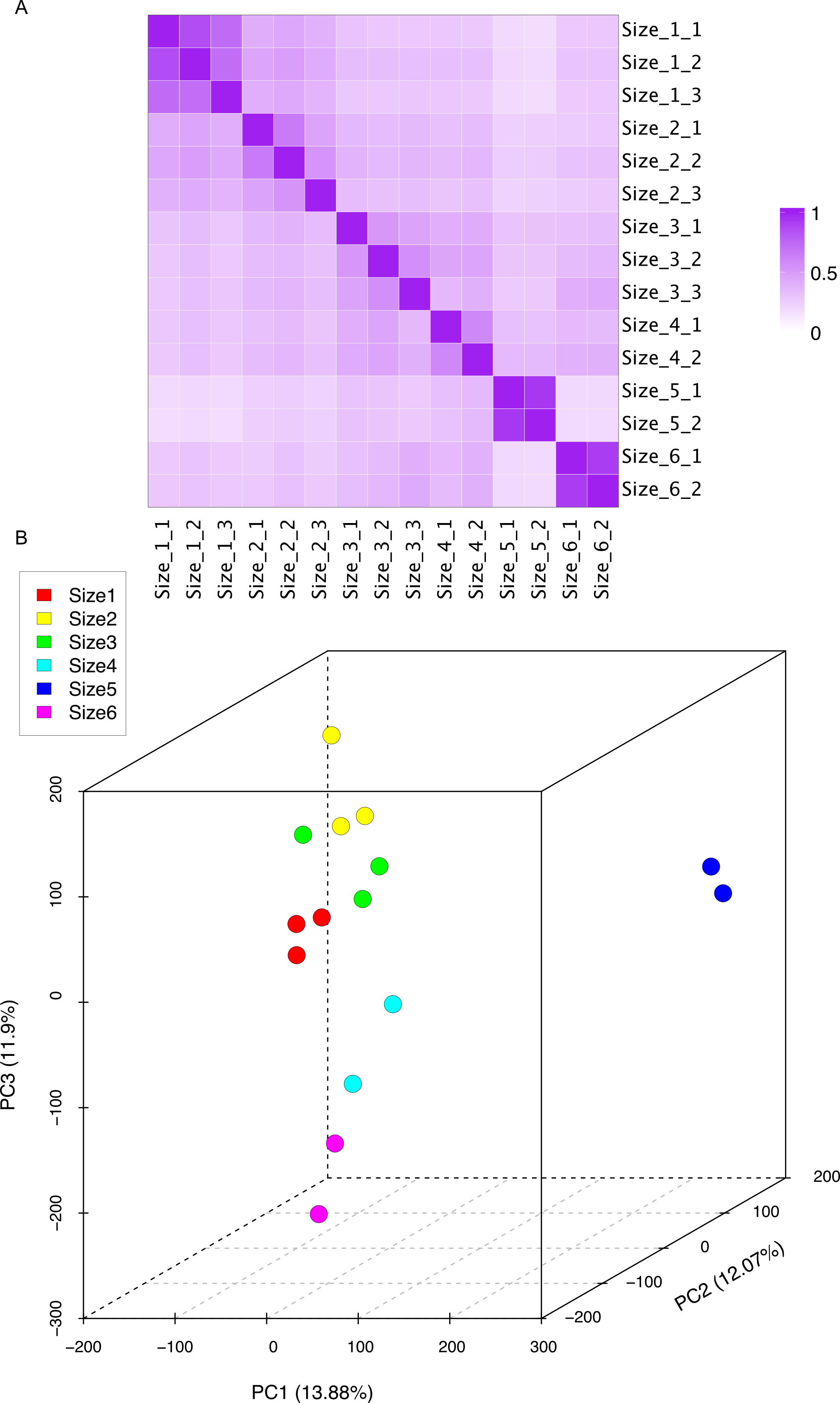

Supplement: Supplementary file 6 [file Image_6.jpg]

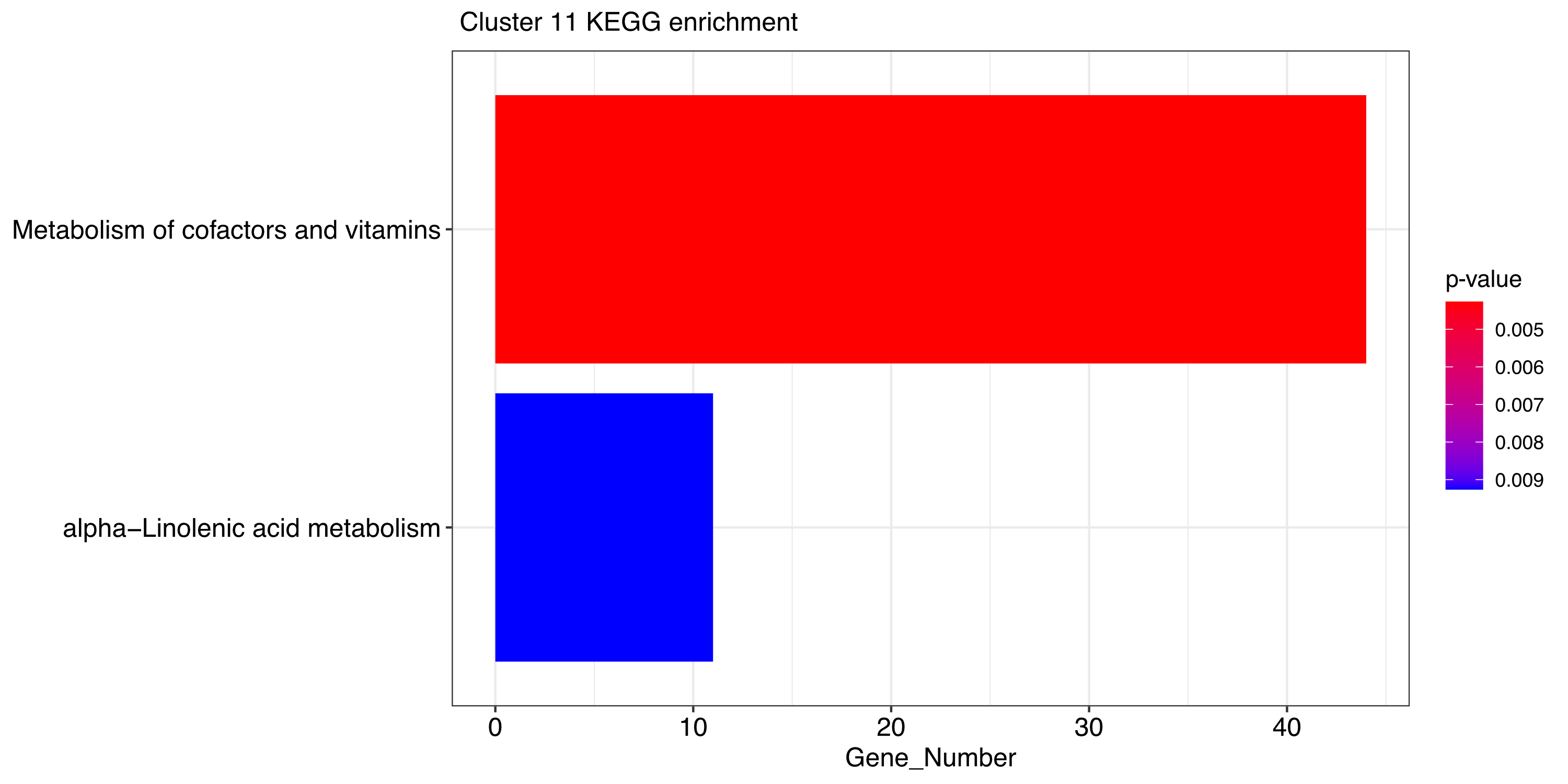

Supplement: Supplementary file 7 [file Image_7.jpg]
